# Demographic Determinants for Change in Activities of Daily Living : A Cohort Study of the Elderly People in Beijing

**DOI:** 10.2188/jea.12.280

**Published:** 2007-11-30

**Authors:** Jingmei Jiang, Zhe Tang, Xiang Jun Meng, Makoto Futatsuka

**Affiliations:** 1 Department of Public Health, Kumamoto University, School of Medicine, Japan.; 2 Beijing Geriatric Clinical and Research Center , Beijing ,China.

**Keywords:** ADL, age-period-cohort analysis, elderly

## Abstract

To describe changes in activities of daily living (ADL) of community-dwelling Beijing elderly people, observed for 8 years, and to identify the demographic characteristics that predict the functional change. Four sets of interview data from1992 to 2000 were used to evaluate changes among Beijing elderly aged 55 years and over. Results revealed that prevalence of disability increased from 3.9% to 7.1% during the 8 years of follow-up with the average increasing rate of disability was 0.41% per year. Meanwhile an increasing likelihood of recovery from disability is observed with age and time. Women, aged 75 or more, experienced higher disability than men though it was in the opposite for younger ages. In addition, certain demographic subgroups (such as women, unmarried, illiterate and living in non urban area) appeared to be at higher risk for ADL impaired. The patterns of ADL change is in both the direction of improvement and declination. Demographic variables emerged as a significant predictor in estimating functional outcomes. Furthermore, it is recommended that the demarcation factor for the evaluation of ADL should be 75 years of age.

## INTRODUCTION

The consequences of decline in mortality for geriatric health have been intensely debated for many years. Crimmins (1989)^1^^)^; Verbrugge (1984)^2^^)^, and Kramen (1983)^3^^)^ suggested that postponement of death gives rise to more chronic diseases and disability. Evidence from the analysis of more recent studies, such as Manton, Corder and Stallard (1997)^4^^)^, and Freedman (1998)^5^^)^ showed decline in disability at older ages, especially for more aged old people whereas work by Crimmins (1997)^6^^,^^7^^)^, EileenM (1997)^8^^)^ yielded mixed results.

Disability refers to an individual’s ability to perform activities of daily living (ADLs) as a reflection of the relationship between personal resources and environmental demands. Verbrugge and Jette (1994)^9^^)^ have offered a model of disability known as the disablement process. This model describes disability as a gap between personal and environmental factors rather than as a personal characteristic. Under this paradigm a person is described as disabled if environment demands exceed his or her personal capabilities.

At any given time, the proportion of elderly disability in population is determined by past rates of prevalence of disability, past rates of restoration in functioning and the history of death rates of the disabled persons in the population^8^^)^. This study examines changes over 8 years of follow up (1992 through 2000) in the prevalence of disability among the elderly in Beijing. Four questions are addressed (1) change during the 1992-2000 in the prevalence of disability, (2) likelihood of elderly people becoming disabled, (3) likelihood of recovery from disability, and (4) likelihood of death during disability. Such measures are however, highly influenced by socially defined roles and the sociocultural and physical environment. So, disability rate is explicitly changed in the composition of the elderly population in a number of demographic characteristics. The roles of demographic in improvement, stability or decline in ADLs functioning are also investigated. Thus, evidence is being looked for change in the prevalence of disability in the elderly population and in the process examine the influence of demographic characteristics that affect this level of disability.

As the number of elderly Chinese grows at an unprecedented rate, there has been increasing concern over the swelling disabled population. So documenting trends in disability and understanding their causes become mandatory for individuals, families, societies, and government to plan for providing health care and long-term care in the future, and promoting independence and delay disability in very late life of individuals or citizens.

## SUBJESTS AND METHODS

### Study Population

The Beijing Longitudinal Study of Aging (BLSA) is a longitudinal investigation, conducted by Beijing Geriatric Clinical and Research Center, of lifestyle, chronic diseases, health in elderly and health care utilization. In the autumn of 1992, a random sample of all community-dwelling persons aged 55 years and over was recruited (the response rate 91.2%). In the autumn of 1994, 1997, and 2000, all survivors of this cohort were contacted for reexamination (Table1).

**Table 1.** Participants in each follow-up survey of the Beijing elderly.

	1992	……	1994	……	1997	……	2000
Invited (survivors of original cohort), no.	3257		2703		2002		1578
Dead between two interviews, no.		384		400		294	
Missing (refused or moved from Interview area), no.		170		301		130	

No. (elderly who participated in all surveys)	1578		1578		1578		1578

BLSA sample consisted of three parts, which covered different residential conditions in Beijing: One representing an urban area, and the rest representing suburban and hilly area respectively. The sample was stratified in terms of sex and age, from 55 to 75 years (in five years cohorts) and over 80, forming six age groups of approximately equal size. The distribution of educational categories of the final sample was found to be consistent with that of older population in Bejing obtained from the Fourth National Census Data.

In this study, the models were stratified by broad age groups (55-64, 65-74, and 75 and over) considering statistical stability in constitution changes after 8 years follow-up.

### Procedures and Measurements

The BLSA cohort study was performed for the total group (all elderly people who participated in at least one survey). At each assessment period interviews were conducted by two trained medical students in the subject’s place of residence. Interviews consisted of a comprehensive biomedical and behavioral assessment, including tests of cognitive functioning, self-report inventories, and ADL scales. In addition, demographic and socioeconomic information were also obtained. Finally, measurements were taken of blood pressure, pulse, grip strength, vision, and hearing.

The main parameter of this study is self-reported physical disability as assessed by the subject’s difficulty in performing ADLs. All of the items were drawn from widely used standardized assessments of these abilities (Kaze, Ford, 1963; Lawton, 1971).

*ADLs* – Many different inventories of activities of daily living exist. The configuration used in the BLSA includes the tasks of walking, bathing, dressing, getting in or out of bed or chairs, grooming, and feeding. Subjects were asked about the amount of difficulty they had in performing the task by themselves without assistance. Each item was scored on a 3-point Likert scale ranging from no difficulty in performing the activity, difficulty, and not being able to perform the activity at all. Disability for a specific activity is defined as the difficulty or inability to do at least one task.

*Sociodemographic Characteristics* – From the information obtained at baseline, we selected the following variables for their possible relation to stability: age, gender, marital status, education and residential status. Marital status were treated as a dichotomous variable: (1) currently married, and (2) unmarried. The unmarried group includes separated, widowed, divorced individuals and as well as those who never married. A dichotomy was used of the relatively small numbers in all these categories (except widowed), Educational attainment was divided into three categories: illiterate, primary, and secondary and above.

### Statistical Methods

All analyses used the Statistical Package for the Social Science (SPSS, version 10.0), A P value of .05 or less was considered to be statistically significant. Changes were assessed in the prevalence of disability with *χ*^2^ tests for independence. The average increasing rate of disability was estimated with ordinary least squares regression models.

Transitions in ADL were analyzed according to three perspectives:

(1) by a longitudinal way: Comparison of the disability status of the elderly was conducted in two interviews and among three transition intervals –(a) from 1992 to 1994 (b) from 1994 to 1997, and (c) from 1997 to 2000. All transitions were analysed in three directions: improvement (restoration from disability), stability (disability continued without improvement), and declination (from ability to disability).

(2) by a cross-sectional way: Comparison of the disability status was conducted in different age groups and different demographic subgroups within each year of study.

(3) by time series: Comparison of the disability status of similar age groups was conducted among different years of the study. Further, a review of disability was conducted for the elderly who did not survive until the second interview.

Considering 33.1% of study population demised during 8-years follow up, their disabled status and demographic characteristics, before death, were also analysed.

## RESULTS

The compositional changes among each survey are shown in Table2. Generally, the change is not significant among each stratified group, except educational attainment of population, which was particularly noteworthy (the proportion of illiterate decreased 7.6% from 51.1% in 1992 to 43.5% in 2000). Table2 and Table3 highlight the need to factor compositional changes out of functional limitation trends within a multivariate framework.

**Table 2.** Constitution changes (as weighted %) in each follow-up surveys.

	1992 ( n=3257 )	1994 ( n=2703 )	1997( n=2002 )	2000 ( n=1578 )
Mean Age, y	70.13	70.99	72.5	74.2
Age range, y	55 – 97	57 – 98	60 – 97	63 – 100
Female,% **	51.1	51.6	51.9	52.1
Region,% **				
Urban	65.6	66.4	65.6	68.0
Suburban	21.1	20.9	20.7	13.1
Hilly	13.3	12.7	13.7	18.9
Marital Status,%				
Married	65.5	65.2	65.8	64.4
Unmarried	34.5	34.8	34.2	35.6
Education,% **				
Illiterate	51.1	49.0	46.4	43.5
Primary	27.1	28.0	29.5	30.9
Second and above	21.7	23.0	24.1	25.6

** Compared among each stratified group. P< .01

**Table 3.** Results of cohort analyses: changes † in activities of daily living(%) between two interviews among Beijing elderly:

	1992(n=3257)	Change in 1994 (n=2703)			Change in 1997 (n=2002)			Change in 2000 (n=1581)		
		
	Baseline	Imp ‡	Stable §	Dec ¶	Imp ‡	Stable §	Dec ¶	Imp ‡	Stable §	Dec ¶
Total	3.9	0.6	1.1	3.9	0.7	1.5	5.1	1.5	1.8	5.3
Age group										
55-64	0.7	0.1	0.3	1.5**	0.4	0.9	2.0 **	1.2	1.0	2.5 **
65-74	2.7	0.5	0.7	3.2	0.9	1.3	3.6	0.3	1.5	6.1
75-	8.0	1.3	2.7	7.8	0.9	3.1	14.2	4.9	5.3	11.5
Sex										
Male	3.3	0.4	0.6	3.3**	0.6	1.4	5.1	1.3	1.7	4.8
Female	4.4	0.8	1.6	4.5	0.8	1.5	5.2	1.6	1.9	5.7
Region										
Urban	3.4	0.6	1.2	3.2	0.6	1.0	4.3 **	1.3	2.0	4.4**
Suburban	5.2	0.7	1.4	5.5	0.7	2.4	8.9	2.0	1.7	4.8
Hilly	3.9	0.3	0.3	5.1	1.1	2.6	3.6	1.5	1.0	8.0
Marital Status										
Married	2.3	0.5	0.6	3.0**	0.5	1.4	3.4 **	1.3	1.4	3.9 **
Unmarried	6.8	0.9	2.2	5.6	1.2	1.8	8.5	1.8	2.7	7.7
Education										
Illiterate	1.4	1.0	1.6	5.1**	0.9	2.3	6.6 **	1.6	2.0	7.0
Primary	3.1	0.4	0.7	3.0	0.3	0.7	3.7	1.4	1.4	4.5
≥ Secondary	5.3	0.0	0.8	2.6	0.8	1.0	4.1	1.2	2.0	3.2

† : changes means transition in ADLs from one statue to another statue between two interviews.

‡ : Improvement (restoration from disability compared the first interview ); § : stability (disability continued without improvement)

¶ : declination (from ability to disability).

** Compared among each stratified group within each year, *χ*^2^ test P< .01.

Generally, the prevalence of disability increased over the 8 years of follow up. At the last three interviews, the likelihood of having an ADL disability is significantly higher than 1992 with the total disability rate as 3.9% (1992), 5.0% (1994), 6.6% (1997), and 7.1% (2000). The average increasing rate of disability for the 1992 sample was 0.41% per year. Taking gender into account, the disability rate was 3.3%, 3.9%, 6.4%, 6.5% for male and 4.4 %, 6.2 %, 6.8 %, 7.7 % for female respectively. Gender average increasing rate was 0.45% per year for men and 0.38% per year for women.

The results of the change of the effect of time interval on the rate of making transitions from “disability to ability”, “ability to disability” and “continued disability” are shown in Table3. All the three total transition rates are observed to increase on the 1992-2000 interval. This is highly encouraging as the rate of overcoming disability increases with years for the elderly people. Although this is a smaller population, it is important to note that it improved the functional status. On the contrary, an increasing disability trend among elders is also seen in the stability and new disability (declination) subgroups and in particular for those aged 75 and more. These results are also confirmed by the cross-sectional analyses.

Most compositional subgroups experienced increasing trends though the levels of transition varied considerably among them. Within each interview, the highest prevalence of new disability and stability is among the women, the unmarried, and the illiterate. Regional differences are also evident.

The changes in direction of most variables showed almost no difference in both longitudinal and cross-sectional studies.

In the time-series perspective (Tables 4), the age was decomposed into two age segments of the young old (65-74) and old old (75 and over). In total disability rate, both groups have increasing trends except a slight decrease in 2000, but the magnitude increase among two groups is different. The average increasing rate was 0.16% per year for the young old and 0.48% per year for the old old. In terms of gender, it is evident that for young old group, women consistently experienced lower disability rate than men except in 1992. However, in old old group, it was opposite with women consistently experiencing higher disability rate than men. Nevertheless, most compositional subgroups experienced increasing trends but with considerable level variations. it is observed that the highest prevalence of disability occurred among the suburban, the unmarried, and the illiterate, and the level of prevalence shows the same trend as in 1992.

**Table 4.** Results of time series analyses: prevalence of disability (%) among four interviews, by age group.

	Ages 65-74				Ages 75 and older			
	
	1992	1994	1997	2000	1992	1994	1997	2000
	(n=1109)	(n=981)	(n=844)	(n=754)	(n=1109)	(n=948)	(n=780)	(n=732)
Total	2.7	3.8	4.5	4.0	8.0	9.3	11.7	11.0
Mean Age, y	69.5	69.6	69.4	69.2	80.4	80.7	80.7	80.8
Sex								
Male	2.1	4.5	5.7	5.0	6.6	5.0**	9.8**	8.3* *
Female	3.4	3.1	3.4	3.1	9.3	13.3	13.5	13.6
Region								
Urban	2.0	3.2	4.2	4.4	7.5	8.4	8.9**	10.3
Suburban	4.8	5.0	4.7	4.1	9.4	11.7	21.3	16.0
Hilly	2.8	4.7	5.5	2.6	8.5	9.7	10.2	9.8
Marital Status								
Married	2.2	3.1	4.8	3.7	6.3	6.5**	8.1**	8.5**
Unmarried	3.9	4.0	3.3	4.8	9.0	11.0	14.3	13.2
Education								
Illiterate	3.3	3.8	4.8	4.3	8.8	10.7	13.8*	13.0 *
Primary	2.7	3.4	4.3	4.0	7.3	8.3	7.1	9.6
≥ Secondary	1.5	4.1	4.3	3.7	4.3	6.1	11.1	7.4

* Compared among each stratified group within each year, P< .05.

** Compared among each stratified group within each year, P< .01.

The mortality rate increased with the increasing age, but there is considerable level variations in most of the compositional subgroups (Table5). The highest mortality, similar to the highest disability rate, occurred among the suburban, the unmarried, and the illiterate.

**Table 5.** A review of disability (%) among elderly who demised between two interviews.

	1992 – 1994				1995 – 1997				1998 – 2000			
		
	No,	( %)†	Prop ‡	Prop ¶	No,	( %)†	Prop ‡	Prop ¶	No,	( %)†	Prop ‡	Prop ¶
Total	384	(11.8)	57.1	18.8	400	(14.8)	57.7	19.8	294	(14.7)	49.6	22.4
Mean Age, Range	77.3 (55 – 97)				77.6 (57 – 98)				77.8 (60 – 96)			
Age												
55-64	32	( 3.1)**	42.9	9.4**	50	( 5.2)**	47.1	16.0	52	( 6.3)**	17.4 *	7.7*
65-74	102	( 9.2)	56.7	16.7	106	(11.0)	44.7	16.0	106	(14.0)	54.1	18.9
75-	250	(22.5)	58.4	20.8	244	(31.4)	65.9	22.1	136	(32.1)	57.5	30.9
Sex												
Male	213	(13.4)**	67.9*	16.9**	208	(15.9)	45.1*	11.1**	146	(15.2)	50.0	21.2
Female	171	(10.3)	49.3	21.1	192	(13.8)	65.1	29.2	148	(14.2)	49.3	23.6
Region												
Urban	208	( 9.7)**	46.6**	16.3	226	(16.8)**	57.5	20.4	150	(11.4)**	39.1*	18.0
Suburban	99	(14.4)	63.9	23.2	118	(20.9)	59.5	18.6	94	(22.7)	66.0	33.0
Hilly	77	(17.7)	88.2	19.5	56	(12.6)	55.0	18.6	50	(18.2)	47.1	16.0
Marital Status												
Married	178	( 8.3)	56.0	15.7	176	( 9.9)**	50.8	18.2	132	(10.0)**	39.7*	18.9
Unmarried	206	(18.3)**	57.9	21.4	224	(23.8)	63.5	21.0	162	(23.6)	58.6	25.3
Education												
Illiterate	251	(15.1)**	57.3	20.3	252	(19.0)**	58.0	20.2	183	(19.7) **	58.5*	26.2
Primary	87	( 9.8)	59.3	18.4	98	(12.9)	67.9	19.4	69	(11.7)	34.6	13.0
≥ Second	46	( 6.5)	50.0	10.9	50	( 8.1)	42.9	18.0	42	( 8.7)	36.0	21.4

† : mortality with study population in the first interview.

‡ : Proportion: Disable elderly who demised between two interviews compared with all disable elderly in the first interview.

¶ : Proportion of disability in all death persons between two interviews;

* ,**: Compared among each stratified group within each status, * P< .05 ; ** P< .01.

The proportion of disability among the demised elderly is not too high (varying between 7.7% to 33.0%), However, high death rates were observed for the disabled in the three time interviews, more than half of disabled elderly could not survive until the next interview, especially in suburban area, unmarried, and the illiterate subgroups. From Table3 & Table5, It is concluded that demographic factors influence the disability rate and the mortality rate significantly.

## DISCUSSION

This study described patterns of improvement, stability and declination in the ADL-management Beijing elderly population. In general, a large proportion of the Beijing elderly are found to remain active in their functional status. Although the disability rate is found to increase with age and with time over 8 years of follow-up, the rate of change is slow especially among those who are younger than the age of 75.

Although a strong age related declination is indicative (e.g Table 3. at the baseline, the disability rate ranged from 0.7% at age group of 55-74 to 8.0% at age group of 75 and over) the deterioration in functional status of the population of elderly, in this investigation, cannot be solely attributed to aging. The increase of prevalence of disability which did not depend on aging is supported by a time series effect. In the time-series analyses, total age-adjusted prevalence of disability continued increasing from 1992 to 1997 for both young old and old old, but it showed a decline in 2000. This decline seemed to be due to a secular trend.

Several studies^10^^-^^12^^)^ have shown that ADL functioning, unlike many other bodily systems, does not follow a consistent pattern of decline but is rather influenced by physical, psychological, and social factors. Our study concurs with these results that elderly people, irrespective of age, could improve in functional status with an increased likelihood of recovery from disability.

Although some studies^13^^-^^14^^)^ have indicated the trend that old women are likely to be physically inactive than men, our studies revealed some variations. Women, only in their later years (aged 75 and over), experienced significantly higher rates of impairment than men, and this trend remained the same in each follow-up survey. Some explanations may contribute to these difference. Our study revealed (data not shown) that women, both young old and old old, also experienced lower death rate than men . Hence, higher life expectancy among women results in higher mean age of disability compared with men. In this study, it is about more than three years older in the mean age among women than that among men.

In addition, demographic variables emerged as a significant predictor in estimating functional outcomes especially in old old elderly. Certain demographic subgroups (such as women, unmarried (about 80% were widowed), illiterate, and those living in non urban area) appeared to be at high risk for physical inactivity and all these results were strongly confirmed by longitudinal, cross-sectional, and time-series analyses.

While interpreting the changes in the functional status of the elderly, one should take into account the effects of selective dropout due to death and nonresponse. as functional status is predictive of mortality 15. Elderly people who did not survive until the end of the follow-up period were less healthy at the baseline (except for oldest old people). Our study revealed that high disability rate accompanies high mortality rate. More than half of the disabled people in the first interview could not survive until the second interview, and the difference is high among the demographic subgroups. For instance, of the all disabled people who were interviewed at the baseline, 88.2% of disabled elderly living in the hilly area did not survive until 1994 which is more than 41.6 % higher than those who were living in the urban area (46.6%). Similarly, there was an obvious difference between gender, marital status, and educational attainment for death rate among disabled. Higher death rate among the disabled people occurred in women, unmarried and illiterate than men, married and those with longer schooling years respectively.

Furthermore our study of disabled elderly is limited by the use of self-reported vs observed disability. These self-reports may reflect morbidity and cognition as they relate to disability. Also, disability for a specific activity in this study is defined as difficulty in performing that function. However, this definition is highly subjective and qualitative as one of the major problems with these definitions are that the receipt of assistance is highly related to the living arrangements and hence those who live with others are more likely to get assistance. In China, more elderly live with a big family. So, when we employed the definition of use-of-assistance, we presumably estimated the disability rate higher than in real life situation.

Although we have not discussed the burden of disability on those who are preparing for the aging of the population in the future, the longitudinal pattern of disability provides insight into the natural history of the progression of disability and has important public health implications. Considering the sharp decline of the resources of the elderly to the steep increase in their environment need, it is recommended that government should organize and develop more public health care schemes to help elderly who are in at higher risk of disability.
